# Nursing education and beliefs towards tobacco cessation and control: a cross- sectional national survey (GHPSS) among nursing students in Greece

**DOI:** 10.1186/1617-9625-9-4

**Published:** 2011-05-06

**Authors:** Evridiki Patelarou, Constantine I Vardavas, Penelope Ntzilepi, Charles W Warren, Anastasia Barbouni, Jenny Kremastinou, Gregory N Connolly, Panagiotis Behrakis

**Affiliations:** 1 University Hospital of Heraklion, Crete, Greece; 2 Department of Social Medicine, School of Medicine, University of Crete, Greece; 3 Center for Global Tobacco Control, Department of Society, Human Development and Health, Harvard School of Public Health, Boston MA, USA; 4 Smoking and Lung Cancer Research Center, Hellenic Anticancer Society, Athens, Greece; 5 Centers for Disease Control and Prevention, Office on Smoking and Health, Global Tobacco Control Program, Atlanta, GA, USA; 6 National School of Public Health, Athens, Greece; 7 School of Medicine, University of Athens, Greece

## Abstract

**Background:**

Within the healthcare system, nurses have the ability to influence their patients' smoking habits through counselling. Therefore, it is of great importance to appropriately train health professionals on smoking cessation strategies with the aim to help them provide advice to their patients. In light of the above, the objective of this study was to assess the association between Greek nursing students' beliefs towards tobacco control/smoking cessation and the professional training received.

**Methods:**

During February 2009, we conducted a cross sectional national survey among all 3^rd ^year nursing students of the two university based nursing departments in Greece (University of Athens, University of the Peloponnese). The Global Health Professional Student Survey (GHPSS) questionnaire was applied and following written informed consent 73% provided a completed questionnaire (n = 192/263 enrolled students).

**Results:**

Overall, 33% were current active smokers, while 74% reported ever to experiment smoking. In regards to their beliefs towards tobacco control policies, non smokers were more positive in regards to banning smoking in restaurants (94% vs. 61%, p < 0.001), in bars and cafes (82% vs. 34%, p < 0.001), and all public places (93% vs. 51%, p < 0.001) when compared to current smokers. In comparison with students who had not received training on the importance of asking patients about their smoking habits, those that did were more likely to believe that nurses should have a role in smoking cessation and should act as role models for their patients.

**Conclusions:**

Resources should be invested in improving the quality of undergraduate education in nursing departments in Greece with respect to tobacco control and smoking cessation.

## Background

Tobacco use is included as one of the leading causes of death and disability worldwide and as it is estimated that almost 1.3 billion people are currently smokers, tobacco use is expected to be the cause of death of about 10 million people per year by 2030 [1].

Despite the above grim projections, health professionals, such as nurses, can play a critical role in tobacco control by providing effective interventions against tobacco use by counseling their patients to quit smoking [2-5]. For the above to be facilitated, it is essential for health care facilities, educational institutes as also medical and nursing associations to acknowledge this role and assume a greater responsibility in promoting smoke-free facilities and practices among their staff and patients [6]. A main barrier in achieving this goal is the increased prevalence of smoking among physicians and nurses in a number of countries. Research has indicated that in several European countries and in USA the prevalence of smoking among physicians has decreased over the past decades, however, in several southern European countries, as well as in countries with transitional economies, the prevalence of tobacco use remains very high [7-9]. In countries with elevated prevalence rates in tobacco consumption a reduction in physician smoking patterns has been shown to be followed by a decrease in the overall population [4]. This fact is of great interest, especially in countries such as Greece, where the tobacco consumption rates are high [6], because health care professionals could act as role models and provide society with a positive example in regards to smoking prevention and cessation [9]. Among others, the existing scientific literature has identified a number of barriers, which include the competing demands and time pressure during patient contacts, the lack of familiarity with effective treatments, perceptions of low receptivity to tobacco interventions, and the absence of adequate training or reimbursement for providing treatment [10-12]. |Moreover the health professional students' own beliefs regarding tobacco cessation and their self-efficacy in providing effective interventions may be major barriers that affect the nurses' role in tobacco cessation [13]. As reported by Sarrna et al. the lack of education among nurses regarding tobacco control is a huge barrier for implementing cessation strategies, to which the World Health Organisation (WHO) has recommended the inclusion of tobacco control in all health professionals' curricula [2].

In light of the above, the main objectives of this study were: 1. To assess the prevalence of smoking among nursing students in Greece and their beliefs towards tobacco and, 2. to assess the interaction between their received undergraduate education on tobacco control and their perception of their role in patient smoking cessation.

## Methods

### Participants and materials

The Global Health Professional Student Survey (GHPSS) has been repeated among third-year students attending dental, medical, and nursing and pharmacy schools in a number of countries and settings [14]. This section of the GHPSS in Greece was conducted during February 2009, within the two university nursing schools that exist in Greece (one in Athens and the other in Sparta) among third year students (In Greece the university degree in nursing is completed after 4 years of training and studies) [15]. At the time of data collection, the total population of enrolled students in the 3^rd ^year of nursing in both departments was 263 students. During this cross sectional survey, the students were asked to complete an anonymous, self-administered questionnaire which was previously translated and back translated into Greek. The questionnaire instrument included information regarding the students main socio- demographic characteristics, smoking habits, attitudes and beliefs toward tobacco control activities and the educational training received in regards to smoking and smoking cessation.

All students were informed about the main aims of the study and consecutively were asked to provide written informed consent, so as to participate in the study. Ethical clearance was provided by the Ethics Committee of University Hospital of Heraklion (Ethics protocol: 106/9-1-2009).

### Statistical analysis

Descriptive results are presented as % (n), while the uivariate analyses for qualitative variables were carried out with the use of x^2 ^tests, with a p < 0.05 classified as statistically significant. Logistic regression models were constructed to examine the role of the participants training on tobacco control in regards to their attitude towards smoking cessation after controlling for the participants smoking status, while odds ratios and 95% Confidence Intervals (95%C.I) were also calculated. The statistical analysis was performed with the statistical package STATA 10.0.

## Results

### Description of participants and smoking status

The final study sample consisted of 192 3^rd ^year nursing students. The total response rate reached the 73% (n = 192/263) of the students totally enrolled. Among the total population, 86.3% were females and 13.7% males. The majority of the respondents were 19-24 years old (93.1%), while only 6.9% were >25 years old. In regards to their smoking habits, 33.1% were current smokers (defined as smoking cigarettes daily or occasionally within the past month), while 74.1% reported experimenting with cigarettes in the past.

### Beliefs and attitudes towards tobacco control by smoking status

Table 1 summarizes the findings regarding the students' attitudes toward tobacco control by smoking status. Almost half of the sample (41%) reported that there was no policy that forbids smoking on campus indoors despite the existence of such legislation, while among those that reported that such a policy existed, the majority (75%) stated that this policy was not enforced. On the whole, a smaller percentage of smokers believed that a policy which forbids smoking indoors on campus exists, when compared to non smokers (54% vs. 71%) and that this policy is enforced (20% vs. 38%) and these differences were statistically significant (*p *< 0.05). In regards to their beliefs towards tobacco control policies, non smokers were more positive in regards to banning smoking in restaurants (94% vs. 61%, p < 0.001), in bars and cafes (82% vs. 34%, p < 0.001), and all public places (93% vs. 51%, p < 0.001) when compared to smokers. (Table 1).

**Table 1:** Greek nursing students' attitudes towards tobacco control and smoking cessation by their smoking status

	Total	Smokers	Non smokers	**p-value**^**1**^
	% (n)	% (n)	% (n)	
Is there any policy that forbids smoking indoors on campus? *(yes)*	59.0 (111)	70.5 (43)	53.5 (68)	**0.027**
Is the above policy enforced? *(yes)*	25.5 (48)	37.7 (23)	19.7 (25)	**0.025**
Should tobacco sales to adolescents be forbidden? *(yes)*	87.4 (166)	85.7 (54)	88.1 (111)	0.643
Should tobacco advertisements totally forbidden? *(yes)*	81.0 (153)	74.6 (47)	84.0 (105)	0.122
Should tobacco smoking in restaurants be forbidden? *(yes)*	82.5 (156)	61.3 (38)	93.7 (118)	**<0.001**
Should tobacco smoking in bars and cafes be forbidden? *(yes)*	65.6 (124)	33.9 (21)	81.8 (103)	**<0.001**
Should tobacco smoking in all enclosed public places be forbidden? *(yes)*	79.1 (148)	50.8 (31)	92.8 (116)	**<0.001**
Should health professionals receive formal training on smoking cessation? *(yes)*	95.3 (182)	93.6 (59)	96.1 (122)	0.461
Should health professionals act as role models for both patients and the public? *(yes)*	91.6 (175)	93.7 (59)	90.6 (115)	0.469
Should health professionals' advise their patients to quit smoking? *(yes)*	97.4 (184)	98.4 (60)	96.9 (123)	0.547
Do health professionals play a role in smoking cessation? *(yes)*	96.9 (185)	96.8 (61)	96.9 (123)	0.993

1: P-values based on two sided tests.

In regards to their received education on tobacco control and smoking cessation, almost all of the participants (more than 90%) agreed that health professionals should receive formal training on smoking cessation (97.4%), should act as role models to their patients (91.6%), and should provide advice so as to promote smoking cessation within the hospital setting (97.4%), with no significant differences by smoking status identified.

### Formal educational training on tobacco control and smoking cessation

Detailed information in regards to the students' beliefs on the training they have received on smoking related issues is depicted in Table 2. The main areas of inadequate training include the reasons that make people start to smoke (less than half of the sample had ever discussed this issue during their studies), smoking cessation techniques (only 14.8% reported ever receiving such training) and the use of antidepressants in smoking cessation (19.5% of the population). No statistically significant differences were identified in the responses by smokers and non smokers, indicating similar levels of training and understanding.

**Table 2:** Greek nursing students' educational background on smoking and smoking cessation (positive responses) by smoking status

During your studies have you ever.....	Total	Smokers	Non smokers	**p-value**^**1**^
	% (n)	% (n)	% (n)	
..received formal training on the dangers of smoking?	91.1 (173)	93.7 (59)	89.8 (114)	0.337
..discussed the reasons why people smoke?	46.8 (89)	38.1 (24)	51.2 (65)	0.089
..been told about the importance of asking about patients' smoking habits?	91.5 (173)	90.3 (56)	92.1 (117)	0.676
..been told about smoking cessation techniques?	14.8 (28)	14.3 (9)	15.1 (19)	0.885
..been told about the importance of counseling material on smoking cessation?	53.4 (101)	47.6 (30)	56.3 (71)	0.257
..been told about nicotine replacement therapies?	93.6 (177)	93.6 (59)	93.6 (118)	1.000
..been told about the use of antidepressants in smoking cessation?	19.5 (370	19.0 (12)	19.7 (25)	0.917

1: P-values based on two sided tests.

Table 3 summarizes the role of formal nursing education on tobacco control on the students' beliefs in regards to their role in providing smoking cessation advice and acting as role models. According to the performed logistic regression analysis and controlling for current smoking status, nursing students were found to be almost six times more likely to believe that they should advise their patients about smoking cessation if they had received previous training on the adverse health affects of smoking and the importance of obtaining a smoking history from the patient (OR 5.7 95% CI: 1.0- 34.2 and 6.0 95%CI: 1.0- 3.6, respectively). Furthermore, students that were taught about the importance of providing material on smoking cessation to patients were more likely to report that nurses have an important role in smoking cessation (OR 2.2 95%CI: 1.0- 4.6) and act as role models towards their patients (OR 2.1 95%CI: 1.7- 6.1). A slight positive association was also revealed between having received training on the importance of providing smoking cessation materials to their patients and the belief that nurses have an advisory and informative role towards their patients, but this association was not statistically significant (OR 2.3 95%CI: 0.4- 13.3).

**Table 3:** Adjusted odds ratio† (OR) and 95% confidence intervals (CIs) for the role of education on nursing students' beliefs towards smoking cessation and training

			Educational aspects		
	**Adverse health effects of smoking**	**Social aspects of smoking**	**Importance of obtaining a smoking history**	**Importance of providing counseling material**	**Use of antidepressants in smoking cessation**
**Beliefs**					
					
**Nurses have a role in smoking cessation**	2.2 (0.7- 6.8)	0.6 (0.3- 1.4)	0.7 (0.2- 3.0)	**2.2 (1.0- 4.6)**	0.6 (0.2- 1.4)
**Nurses should receive special training**	1.4 (0.2- 11.7)	1.1 (0.3- 4.1)	1.6 (0.2- 13.6)	0.5 (0.1- 2.2)	0.8 (0.2- 4.2)
**Nurses should be role models**	1.5 (0.3- 7.0)	1.2 (0.4- 3.4)	1.7 (0.4- 8.0)	**2.1 (1.7- 6.1)**	0.4 (0.1- 1.1)
**Nurse should advise their patients to quit**	2.6 (0.3- 25.1)	1.5 (0.3- 8.9)	2.9 (0.3- 27.8)	1.9 (0.3- 11.5)	0.9 (0.1-8.8)
**Nurses should provide information on smoking cessation**	**5.7 (1.0- 34.2)**	*	**6.0 (1.0- 36)**	2.3 (0.4- 13.3)	*

† adjusted for smoking habits (smoker/non smoker)

* not enough subjects to perform the analysis.

## Discussion

This study revealed a high percentage of nursing students that were smokers in Greece. Furthermore, this study revealed that students' education on smoking cessation and tobacco control was highly associated with their positive perception that they can play a role in tobacco control issues and aid smoking cessation among patients, a fact that indicates the importance of adopting a relative curriculum within the undergraduate nursing curriculum in Greece.

Results of the international GHPSS study conducted among health professional students revealed that a 20% were current smokers, the majority of which believed that they should receive formal training on counseling their patients to quit tobacco, however, less than 40% of the students reported they have received such training during their undergraduate studies [14,16]. Another, recent GHPSS study conducted in Lebanon among health professional students showed that the prevalence of smoking among nursing students was 26.9%, and that 9 in 10 health professional students believed that health professionals should receive training on smoking cessation techniques, percentages similar to those found within the context of our study [17]. In the same study the percentage of nursing students that reported that they had received training on tobacco related issues during their undergraduate studies reached almost 50% [17]. Our study confirmed the evidence that nurses with previous education on smoking cessation and training related issues were more likely to support the idea that nurses do have an advisory and informative role in counseling patients and should promote tobacco control and prevention. There is evidence supporting that nurses who had received training were more likely to perform tobacco control interventions than untrained ones while the absence of comprehensive tobacco prevention and cessation training in health care education can result in lost opportunities for such interventions [18].

It is critical to examine the perceptions towards tobacco control by health students as they form their professional roles and develop their basic practices while at university [19]. Nurses, as the largest group of health care professionals, and due to their role in the health care system spend more direct time with patients, and can perform health promotional activities. Nurses have the advantage that they could provide people with smoking cessation techniques and act as an effective approach to decrease tobacco use [1,13]. It is necessary to put into practice educational programs in nursing schools, on the prevention and treatment of smoking. Our findings indicate an eminent need to improve the nursing curriculum within the area of tobacco control, including information on prevention, policy and dependency as the nursing students who had received formal training were more likely to acknowledge their position as a role model and as a vector for smoking cessation.

In Greece, currently there is a lack in the content of the nursing curriculum in preparing nursing students to provide adequate advice to patients in regards to their smoking habits. Moreover in all medical and nursing departments there is no separate educational module on tobacco control, only a number of separate lectures. Therefore, there is a need to re-examine the curriculum to enable students to diagnose, manage and treat smoking dependency and acknowledge the fundamental aspects of tobacco control. Knowledge on the basic, community based and clinical science related to tobacco use, attitudes and behaviours are minimal skills that should be core graduation requirement for nursing students. In the USA, it has been suggested that nursing students lack training on how to implement tobacco cessation techniques and that increased instructional efforts concerning the clinical treatment of tobacco dependence would be critical in order to achieve a decrease in smoking rates [18,20]. Nursing students' knowledge of intervention techniques and methods to help people give up smoking was found to be poor overall, indicating the need to improve the content of current nursing degrees on the prevention and treatment of tobacco use [21]. Similarly, Sarna et al. concluded that nurses in four Asian countries (China, Korea, Japan and the Philippines) may not be adequately prepared to help tobacco users quit smoking, because the training programs do not offer content related to comprehensive cessation interventions [2,20]. Furthermore, Sekijima et al. [22] mentioned that Japanese students and nursing staff did not consistently receive adequate education for the generation of skills needed to apply smoking cessation within the context of their training [22].

Currently there are already several educational methods and core competencies with clear learning objectives on smoking interventions that can be implemented in nursing schools [23]. Nursing schools need to take action aimed at including approaches to smoking in their curriculum, and also promote the adoption of continuing educational courses for established health care professionals, especially for those who practise within primary care. There is need for the development of a specific curriculum to teach students on how to assist smokers to quit and how to counsel non-smoking adolescents so as to prevent them from starting to smoke. These goals have been achieved in some countries that have a core competency and learning curriculum for tobacco education in medical schools [24], nursing schools could follow.

This study adds evidence to the growing body of knowledge that there is a link between receiving formal education during undergraduate studies on tobacco control and the students' beliefs regarding smoking cessation and their duty as role models towards their patients. Nevertheless, it is important to acknowledge that this was a cross- sectional study and thus it is not possible to make definitive statements regarding causality, while the relatively large confidence intervals can be explained by the relatively small sample size in each subgroup analyzed. On the other hand, the representative sampling frame and standardized procedures of the GHPSS study increase the validity and generalisability of our study's key findings among nursing students, and most likely recent graduates in Greece.

## Conclusions

Educational institutes, public health organizations and education officials in Greece, should discourage tobacco use among health professional students and work together to design and implement programs that train all health professionals on tobacco control and on effective cessation counseling techniques. Nurses are a large and strategically planned occupational group, which could help avert the global tobacco epidemic [25]. As a result, resources should be invested in improving the quality of education of nurses with respect to tobacco control so as to empower them with the capability to successfully implement smoking prevention and cessation techniques during routine clinical practice.

## Authors' contributions

Author EP was responsible for the data analysis, interpretation of the results and had the main role in manuscript preparation. Author CV was responsible for study concept and design and contributed to manuscript preparation, NP and AB participated in study management and manuscript preparation, WW participated in study design and data preparation, PP participated in data collection and manuscript preparation, GC participated in data interpretation and manuscript preparation, while JK and PB participated in study design, result interpretation and manuscript preparation. All authors have read and approved the manuscript.

## Conflict of interest

The authors declare that they have no competing interests.

## Funding

This work was partially supported by the HEART project (Hellenic Action for Research against Tobacco), funded by the Behrakis Foundation through the "Behrakis Project for making smoking history in Greece".
